# A new quantum speed-meter interferometer: measuring speed to search for intermediate mass black holes

**DOI:** 10.1038/s41377-018-0004-2

**Published:** 2018-05-30

**Authors:** Stefan L. Danilishin, Eugene Knyazev, Nikita V. Voronchev, Farid Ya. Khalili, Christian Gräf, Sebastian Steinlechner, Jan-Simon Hennig, Stefan Hild

**Affiliations:** 1 Institut für Theoretische Physik, Leibniz Universität Hannover and Max-Planck-Institut für Gravitationsphysik (Albert-Einstein-Institut), Callinstraße 38, D-30167 Hannover, Germany; 2 SUPA, School of Physics and Astronomy, University of Glasgow, Glasgow, G12 8QQ United Kingdom; 3 M.V. Lomonosov Moscow State University, Faculty of Physics, Moscow, 119991 Russia; 4 Institut für Laserphysik und Zentrum für Optische Quantentechnologien der Universität Hamburg, Luruper Chaussee 149, 22761 Hamburg, Germany

## Abstract

The recent discovery of gravitational waves (GW) by Advanced LIGO (Laser Interferometric Gravitational-wave Observatory) has impressively launched the novel field of gravitational astronomy and allowed us to glimpse exciting objects about which we could previously only speculate. Further sensitivity improvements at the low-frequency end of the detection band of future GW observatories must rely on quantum non-demolition (QND) methods to suppress fundamental quantum fluctuations of the light fields used to readout the GW signal. Here we present a novel concept of how to turn a conventional Michelson interferometer into a QND speed-meter interferometer with coherently suppressed quantum back-action noise. We use two orthogonal polarizations of light and an optical circulator to couple them. We carry out a detailed analysis of how imperfections and optical loss influence the achievable sensitivity. We find that the proposed configuration significantly enhances the low-frequency sensitivity and increases the observable event rate of binary black-hole coalescences in the range of $10^2 - 10^3\,M_ \odot$ by a factor of up to ~300.

## Gravitational wave detection: Improved Sensitivity

A new design of interferometric gravitational wave detector proposed by scientists from Russia, Germany and the UK could potentially boost the detection rate of intermediate black hole events by a factor of several hundred. Analysis indicates that the team’s “polarization circulation speed meter” interferometer design has significantly better noise performance at low frequencies (1-100 Hz) than traditional Fabry-Perot Michelson interferometers. The improvement is due to coherent suppression of quantum back-action noise and the use of two orthogonal polarizations of light. A critical factor in the performance of the design is the optical loss of the quarter wave plate used to separate the two polarizations of light and this needs to be kept as low as possible. The team is now investigating the benefit of using squeezed light states with the scheme for further sensitivity improvements.

## Introduction

The recently reported breakthrough observation of gravitational waves emitted by coalescing binary black holes marked the starting point of the new field of gravitational-wave astronomy^1^. The observations of Advanced LIGO (Laser Interferometric Gravitational-wave Observatory) produced evidence of a new population of black holes not consistent with our previous knowledge based on X-ray observations^2^. Increasing the low-frequency sensitivity of current and future gravitational-wave observatories will not only allow us to improve the signal-to-noise ratio with which we can observe them but also allow us to extend our observation capability to even heavier binary black-hole systems. This will allow us to shed light on many important questions, such as: What is the precise astrophysical production route of binary black-hole systems of tens of solar masses? What is the nature of spin–orbit and spin–spin coupling in coalescing binary black holes? Are the no-hair theorem and the second law of black-hole mechanics valid?

To enhance the low-frequency sensitivity of future gravitational-wave detectors, a variety of noise sources must be addressed and improved, of which the most fundamental is so-called quantum noise, an inherent consequence of the quantum mechanics of the measurement process.

In the late 1960s, Braginskiǐ^3^ identified quantum fluctuations of the electromagnetic field as the main fundamental limitation to the sensitivity of electromagnetic weak force sensors. He showed that continuous monitoring of the test object position to infer an external weak force (e.g., GW) always leads to a quantum back-action of the meter on the probed object’s position, thereby setting the standard quantum limit (SQL) on the achievable precision of such a measurement. In interferometric sensors such as GW interferometers, light is used to monitor the distances between mirrors. Here, back-action noise originates from the quantum fluctuations of the light’s intensity, leading to random radiation-pressure forces acting on the mirrors. The corresponding additional displacement noise is most pronounced at low frequencies due to the mirrors’ dynamical response and stems from the fundamental quantum fluctuations of the light’s phase, setting the imprecision of the position monitoring ($\Delta x_{{\rm imp}} \propto 1/\sqrt {N_{{\rm ph}}}$) (here *N*_ph_ is the number of photons used for the measurement) and the back-action noise ($\Delta x_{{\rm BA}} \propto \sqrt {N_{{\rm ph}}}$). Evidently, the naive trade-off in *N*_ph_ yields the SQL; that is, the point at which $\Delta x_{{\rm imp}} = \Delta x_{{\rm BA}}$.

The SQL stems from non-commutativity of the displacement as an operator at different times, i.e., $[\hat x(t),\,\hat x(t\prime )] \ne 0$, which means that a displacement measurement at time *t* will influence the result of one at a later time *t*′. Observables that commute at different times and thus can be monitored continuously with arbitrary precision are known as quantum non-demolition (QND) observables. The obvious choices for such observables are the conserved quantities of the test object, such as energy, quadratures for the oscillator, or momentum for a free mass.

Velocity measurement as a QND procedure proposed in ref.^4^ is based on the premise that at timescales shorter than the suspension-pendulum period, the mirror behaves as a free mass and its momentum is conserved and proportional to its velocity, $\hat p = m\hat v$. A more careful analysis has shown that the dynamics of the test object cannot be considered separately from that of the meter, which is the laser light in the case of GW interferometers. For a combined system ‘mirrors + light’, the generalized momentum is a sum of two terms, $\hat P = m\hat v - g_{{\rm SM}}(t)\hat a_c$, rather than a simple proportionality to velocity (see, e.g., section. 4.5.2 in ref.^5^), where $g_{{\rm SM}}(t)$ is the strength of coupling between the light and the mirrors’ mechanical motions and $\hat a_c = (\hat a + \hat a^\dagger )/\sqrt 2$ is the amplitude quadrature of the light (defined in terms of photon annihilation (creation) operators $\hat a$ ($\hat a^\dagger$)). Though sensing the mirrors’ velocity via an outgoing light phase-quadrature measurement is not a QND measurement, it nevertheless provides a substantial reduction of random back-action force^5^.

**Fig. 1:**
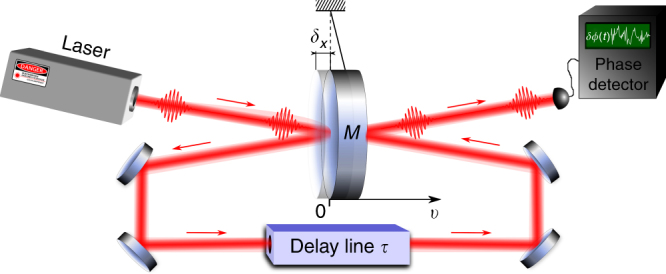
**Conceptual scheme of optical speed measurement with two consecutive reflections of the light pulses from the front and the rear surfaces of the mirror**

The simplest conceptual realization of an optical speed meter is shown in Fig. 1^6^. Here, a laser sends short light pulses to the suspended mirror. The pulses are reflected from the mirror twice with a time delay *τ* between the reflections. After each reflection, the mirror’s displacement is written in the phase of the pulse; hence, after two reflections the pulse’s phase is shifted by $\phi _{{\rm pulse}} \propto \hat x(t+\tau) - \hat x(t) \sim \tau \bar v$, where $\bar v$ is the mean velocity. Note that since the momentums transferred to the mirror by photons in the two reflections have opposite signs, and since there is no decoherence between the reflections, they compensate each other. Therefore, quantum back-action noise is suppressed by $\sim \tau /T_{{\rm signal}} \propto \Omega _{{\rm signal}}\tau$, where $T_{{\rm signal}} = 2\pi /\Omega _{{\rm signal}}$ is the specific timescale of the signal force, e.g., the period of a GW.

This example illustrates the two key features that the measurement scheme should possess to realize a speed measurement: (i) the probe (light) must interact with the test object (mirror) twice, retaining coherence between the interactions (for coherent suppression of back-action noise), and (ii) the two terms in the interaction Hamiltonian that relate to the two consecutive measurements should have opposite signs^7^.

**Fig. 2:**
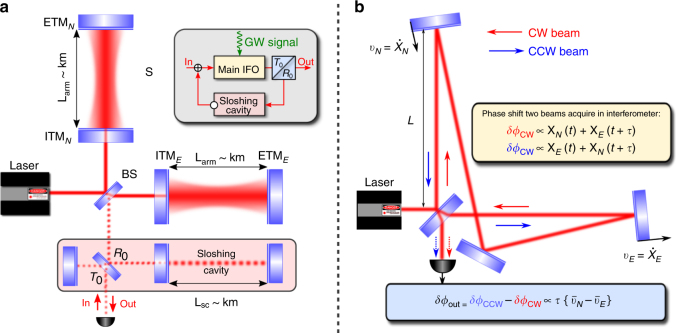
Two variants of speed-meter interferometers. **a** The sloshing speed meter, and **b** the Sagnac speed meter. Inset in **a** is a block diagram of the sloshing speed meter principle of operation. Here (I)ETM stands for (input) end test mass, BS is a beam splitter, and $T_0 = 1 - R_0$ is the (power) transmissivity of the output coupling mirror

The first implementation proposed for detection of gravitational waves was in ref.^7^ (Fig. 2a), where a traditional Fabry–Pérot–Michelson interferometer was extended by an auxiliary “sloshing” optical cavity in the output port. This caused the GW signal to “slosh” back and forth between the two coupled effective cavities with alternating phase. Hence, after the second pass through the interferometer, the outgoing light would bear exactly the required combination of position signals, $\propto \hat x(t+\tau) - \hat x(t) \sim \tau \bar v$, yielding the speed measurement. This scheme was nicknamed a “sloshing speed meter”. It has the distinctive feature that carrier and signal light beams do not share the same optical path throughout the interaction because the sloshing cavity is kept not pumped by a laser. This makes it very difficult to lock and control, and may also lead to signal loss from distortion in optical elements. A practical version of the sloshing speed-meter scheme was analyzed in great detail in refs.^8, 9,]^.

Another solution was proposed by Chen in ref.^10^, demonstrating that a Sagnac interferometer with zero area performs a speed measurement. Here, the double measurement of the mirror position is performed naturally by two counter-propagating light beams, which, after recombination on the beam splitter, produce the signal beam whose phase depends on the mean relative velocity of the end mirrors (see Fig. 2b and ref.^6^ for analysis).

Quantum back-action noise suppression in both schemes depends on the fact that the radiation pressure force component, which drives the differential displacement of the arm mirrors, $x_{{\rm dARM}} = x_n - x_e$, stems from the beat note of the carrier classical amplitude $A \propto \sqrt {P_c}$ (*P*_*c*_ is the laser power circulating in the arms) with vacuum fields, ${\hat{\mathbf i}}$, entering the readout port of the interferometer rather than with the laser fluctuations, $\hat F^{b.a.}(t) \propto A\,\hat i_c(t)$, with $\hat i_c$ the amplitude quadrature of ${\hat{\mathbf i}}$. In sloshing speed meters, the subtraction of two back-action kicks is provided by the *π*-phase shift that the dark port field acquires after the reflection off the sloshing cavity; hence, $\hat F^{b.a.} \propto A\,\hat i_c(t) + e^{i\pi }A\,\hat i_c(t + \tau _{{\rm sl}}) = A\,(\hat i_c(t) - \hat i_c(t + \tau _{{\rm sl}}))$, and *τ*_sl_ is the characteristic time of optical energy sloshing between the coupled cavities of the sloshing speed-meter interferometer.

In a Sagnac interferometer, the required “minus” sign is provided by the phase difference of *π* between the reflected and transmitted beams at the beam splitter. The suppression of quantum back-action here originates from the opposite sign of the radiation pressure forces from the clockwise and counter clockwise propagating light beams, i.e., $\hat F_{{\rm CW}}^{b.a.} + \hat F_{{\rm CCW}}^{b.a.} \propto A\,\hat i_c\left( t \right) - A\,\hat i_c\left( {t + \tau _{{\rm prop}}} \right),$ with *τ*_prop_ the light propagation time between the arms.

The complexity of experimental implementation of these schemes led to the idea of using two orthogonal polarizations of light to separate the two beams sensing the mirrors in a Sagnac-type speed meter^11, 12^. This approach allows keeping the km-scale arm cavities of the original Michelson unchanged, but requires substantial modification to the input and output optics and the implementation of additional polarizing elements of large physical dimensions, which have not yet been used inside the core interferometers.

An alternative scheme, proposed in ref.^13^, makes use of the differential optical mode of the Michelson interferometer with the polarization orthogonal to that of the pumping laser as an effective sloshing cavity. The polarization separation of the signal light fields from the “sloshing” ones is achieved with six optical elements: two quarter wave plates (QWP), 2 mirrors, a polarization beam splitter (PBS) and an additional (omitted in ref.^13^) birefringent plate that flips the sign of the vertically polarized signal sidebands reflected from the “sloshing cavity”.

**Fig. 3:**
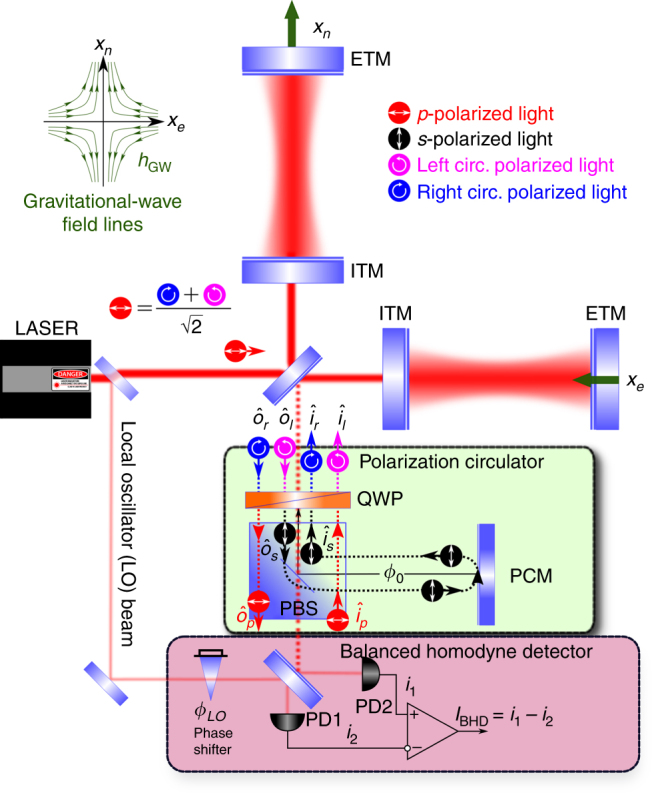
Possible realization of the polarization circulation interferometer, using a quarter-wave plate (QWP) for polarization separation. Here, (E)ITM stands for (end) input test mass, PCM is a polarization circulation mirror, PBS is a polarization beam splitter and PD is a photodetector. Note that the BHD readout setup shown here does not reflect the full complexity of the homodyne readout schemes developed for real GW detectors that have been studied extensively elsewhere^14, 15^

In this letter, we propose a new, even simpler scheme with only 3 extra elements in which the two orthogonal polarization modes of the Michelson interferometer serve as two counter-propagating beams of a Sagnac-type interferometer. The scheme is shown in Fig. 3. The minimum of optical elements involved, as well as relaxed requirements on their position control, makes our speed meter the most robust to loss and imperfections and easiest to implement in the next generation of GW detectors, as we show below.

## Materials and methods

### Polarization circulation interferometer as a speed meter

The main interferometer is pumped by a strong *p*-polarized laser field **p**_*p*_ that can be represented as a linear combination of two circularly polarized fields (marked by $(l)r$ for (counter)clockwise-polarization) with polarization basis vectors, $\vec e_j$ ($j = \{ p,\,r\,,l\}$):

$${\mathbf{p}}_p\vec e_p = {\mathbf{p}}_r\vec e_r + {\mathbf{p}}_l\vec e_l\,,\quad \left| {{\mathbf{p}}_r} \right| = \left| {{\mathbf{p}}_l} \right| = \frac{{\left| {{\mathbf{p}}_p} \right|}}{{\sqrt 2 }}$$

Coherent coupling between the two polarizations is performed by the polarization circulator comprising the QWP, PBS and the closing highly reflective mirror. The PBS and QWP define the new circular polarization basis for the light modes of the interferometer. The PBS passes the *p*-polarized vacuum field, ${\hat{\mathbf i}}_p$, that is transformed by the QWP into the *l*-polarized field ${\hat{\mathbf i}}_l$. This field enters the Michelson interferometer from the dark port and interacts opto-mechanically with the **p**_*l*_ component of the pumping laser field **p** and the differential mechanical degree of freedom of the interferometer mirrors, $x_{{\rm dARM}}(t) = x_n(t) - x_e(t)$. The outgoing *l*-polarized field ${\hat{\mathbf o}}_l$, carrying information about the $x_{{\rm dARM}}$ displacement, is transformed into the *s*-polarized field ${\hat{\mathbf o}}_s$, which is reflected by the PBS toward the polarization circulation mirror (PCM). The latter reflects ${\hat{\mathbf o}}_s$ back toward the PBS, where it arrives with an acquired phase shift $2\phi _0 = \pi$ and enters the main interferometer as ${\hat{\mathbf i}}_r$ after being transformed by the QWP. Delayed by the arm cavity ring-down time *τ*, it senses the $x_{{\rm dARM}}(t + \tau ) = x_n(t + \tau ) - x_e(t + \tau )$ and couples with the **p**_*r*_ component of the pumping laser field **p**.

The *r*-polarized output field ${\hat{\mathbf o}}_r$ leaves the readout port of the interferometer, is transformed by the QWP into the *p*-polarized field ${\hat{\mathbf o}}_p$, and is transmitted by the PBS toward the balanced homodyne detector (BHD). The readout photocurrent is then proportional to the differential speed of the change of the arms’ lengths:

$$I_{{\rm BHD}} \propto x_{{\rm dARM}}(t + \tau ) - x_{{\rm dARM}}(t) \simeq \tau \bar v_{{\rm dARM}}(t)$$

### Quantum-noise-limited sensitivity

To give a more quantitative account of the quantum noise behavior of the proposed scheme, we use the two-photon formalism of quantum optics^16^. In this formalism, the electric field strain of the plane electromagnetic wave of the laser beam with frequency *ω*_*p*_, cross-section area ${\cal A}$ and power *P*_in_ can be written as: $\hat E(t) = {\cal E}_0\left[ {(A + \hat a_c(t)){\mathrm{cos}}\,\omega _pt + \hat a_s(t){\mathrm{sin}}\,\omega _pt} \right]$, where ${\cal E}_0 = \sqrt {4\,\pi \,\hbar \,\omega _p/({\cal A}c)}$ is the second quantization normalizing constant, $A = \sqrt {2P_{{\rm in}}/(\hbar \omega _p)}$ is the carrier dimensionless amplitude, and $\hat a_{c,s}(t)$ are the cosine (“c”) and sine (“s”) quadrature amplitudes of the quantum fluctuations with zero mean.

Here, variations from the mean value of two conjugate quadratures of the light field are given by a 2D vector ${\hat{\mathbf i}} \equiv \{ \hat i_c,\,\hat i_s\} ^T$ of the amplitude and the phase quadrature operators, respectively. Analysis of quantum noise of any interferometer starts from deriving the relations between the input and output light quadrature amplitudes, or I/O-relations for sideband fields at an offset frequency $\Omega = \omega - \omega _p$. For a lossless interferometer tuned in resonance, so that the GW signal appears only in the phase quadrature, the general shape of the I/O-relation is very simple^17^:

1 $$\begin{array}{*{20}{l}} {\hat o_{p,c}\left( \Omega \right)} \hfill & { = e^{2i\beta }\hat i_{p,c}\left( \Omega \right)\,,} \hfill \\ {\hat o_{p,s}\left( \Omega \right)} \hfill & { = e^{2i\beta }\left[ {\hat i_{p,s}\left( \Omega \right) - {\cal K}\hat i_{p,c}\left( \Omega \right)} \right] + e^{i\beta }\sqrt {2{\cal K}} \frac{{h\left( \Omega \right)}}{{h_{{\rm SQL}}}}} \hfill \end{array}$$

where ${\cal K}(\Omega )$ is an optomechanical coupling factor describing the interaction of the mechanical degrees of freedom of the interferometer with light, $\beta (\Omega )$ is the frequency-dependent phase shift acquired by sideband fields as they pass through the interferometer, and $h_{{\rm SQL}} = \sqrt {\frac{{8\hbar }}{{ML^2\Omega ^2}}}$ stands for the GW strain standard quantum limit for the effective mechanical mode of the interferometer with reduced mass *M* and arm length *L*. The second term in the brackets in Eq. (1) originates from the radiation pressure force driven by amplitude fluctuations. The last term in (1) describes the response of the interferometer to the GW signal with strain $h(\Omega ) = 2x_{{\rm dARM}}(\Omega )/L$.

One can then derive the quantum noise power spectral density (PSD) from the above I/O-relations in the following form:

2 $$S^h(\Omega ) = \frac{{h_{{\rm SQL}}^2}}{2}\left\{ {\frac{{1 + [{\cal K}(\Omega ) - {\mathrm{cot}}\phi _{LO}]^2}}{{{\cal K}(\Omega )}}} \right\}$$

where we assumed the homodyne readout of arbitrary quadrature defined by the local oscillator phase $\phi _{LO}$.

The formulae for ${\cal K}$ and *β* for any tuned interferometer configuration can be derived easily, as we show below. For a Michelson interferometer with total circulating power in each arm *P*_*c*_, laser frequency $\omega _p = 2\pi c/\lambda _p$ and effective half-bandwidth *γ*, it is ${\cal K}_{{\rm MI}} = \frac{{2\Theta \gamma }}{{\Omega ^2(\gamma ^2 + \Omega ^2)}}$ with $\Theta \equiv \frac{{8\omega _pP_c}}{{{McL}}}$, and frequency-dependent sideband phase shift $\beta _{{\rm MI}} = {\mathrm{arctan}}\frac{\Omega }{\gamma }$. As shown in the Methods section, the same expression for the polarization circulation interferometer in the speed-meter regime is:

$${\cal K}_{{\rm PCSM}} = 2{\cal K}_{{\rm MI}}{\mathrm{sin}}^2\beta _{{\rm MI}} = \frac{{4\Theta \gamma }}{{(\gamma ^2 + \Omega ^2)^2}}$$

In the more general case of a detuned interferometer, the I/O-relations can be written in matrix notation as follows:

$${\mathbf{o}} = {\Bbb T}{\mathbf{i}} + {\mathbf{t}}\frac{h}{{h_{{\rm SQL}}}},\,{\mathrm{where}}\,{\Bbb T} = \left[ {\begin{array}{*{20}{c}} {T_{cc}} & {T_{cs}} \\ {T_{sc}} & {T_{ss}} \end{array}} \right],\quad {\mathbf{t}} = \left[ {\begin{array}{*{20}{c}} {t_c} \\ {t_s} \end{array}} \right]$$

where ${\mathbf{i}} = [\hat i_c(\Omega ),\,\hat i_s(\Omega )]^T$ and ${\mathbf{o}} = [\hat o_c(\Omega ),\,\hat o_s(\Omega )]^T$ are the two-dimensional (2D) vectors of the input and the output light quadratures, respectively, ${\Bbb T}(\Omega )$ is a 2 × 2-matrix of the corresponding optical transfer matrices for the light fields, and ${\mathbf{t}}(\Omega )$ is a 2D vector of optomechanical response functions that characterizes how a GW with strain amplitude spectrum $h(\Omega )$ shows itself in the output quadratures of the light leaving the interferometer.

The readout photocurrent of the balanced homodyne detector is proportional to the quadrature of the outgoing light defined by the local oscillator phase angle $\phi _{LO}$. Thus we can define the readout quadrature proportional to $\hat I_{{\rm BHD}}(\phi _{LO})$ as:

$$\hat o_{\phi _{LO}} \equiv \hat o_c{\mathrm{cos}}\phi _{LO} + \hat o_s{\mathrm{sin}}\phi _{LO} \equiv {\mathbf{H}}_{\phi _{LO}}^T \cdot {\mathbf{b}}$$

with ${\mathbf{H}}_{\phi _{LO}} \equiv \{ {\mathrm{cos}}\phi _{LO},\,{\mathrm{sin}}\phi _{LO}\} ^T,$ and the spectral density of quantum noise at the output port of the interferometer can be obtained using the following simple rule:

3 $$S^h(\Omega ) = h_{{\rm SQL}}^2\frac{{{\mathbf{H}}_{\phi _{LO}}^T \cdot {\Bbb T} \cdot {\Bbb S}_i^{in} \cdot {\Bbb T}^\dagger \cdot {\mathbf{H}}_{\phi _{LO}}}}{{|{\mathbf{H}}_{\phi _{LO}}^T \cdot {\mathbf{t}}_h|^2}}$$

where ${\Bbb S}_i^{{\rm in}}$ is the spectral density matrix of injected light and its components and can be defined as:

$$\begin{array}{*{20}{c}} {2\pi \delta (\Omega - \Omega \prime )\,{\Bbb S}_{i,\mu \nu }^{{\rm in}}(\Omega ) \equiv } \\ {\frac{1}{2}\left\langle {{\rm in}|\hat a_\mu (\Omega )(\hat a_\nu (\Omega \prime ))^\dagger + (\hat a_\nu (\Omega \prime ))^\dagger \hat a_\mu (\Omega )|{\rm in}} \right\rangle } \end{array}$$

where $|{\rm in}\rangle$ is the quantum state of vacuum injected in the dark port of the interferometer and $(\mu ,\nu ) = \{ c,s\}$ (see section 3.3 in ref.^5^ for more details). In the present article, we address single-sided spectral densities *S* and hence in the case of the input vacuum state:

$$|{\rm in}\rangle = |{\rm vac}\rangle \quad \quad \Rightarrow \quad \quad {\Bbb S}_a^{{\rm in}} = {\Bbb I}$$

### Derivation of I/O-relations of the polarization circulation speed meter

The I/O-relations for our scheme can be obtained using the Michelson interferometer I/O-relations for each of the ±45°-polarization modes. One just needs to keep in mind that both polarizations contribute to the common back-action force. The corresponding transfer matrix ${\Bbb T}_{{\rm MI}}$ and response vector, **t**_MI_ read:

$${\Bbb T}_{{\rm MI}} = e^{2i\beta _{{\rm MI}}}\left[ {\begin{array}{*{20}{c}} 0 & 1 \\ { - {\cal K}_{{\rm MI}}} & 1 \end{array}} \right],\quad {\mathbf{t}}_{{\rm MI}} = e^{i\beta _{{\rm MI}}}\sqrt {2{\cal K}_{{\rm MI}}} \left[ {\begin{array}{*{20}{c}} 0 \\ 1 \end{array}} \right]$$

In the proposed scheme, each polarization mode, **p**_*l*_ and **p**_*r*_, has half of the total circulating power provided by the pump laser. Therefore, each mode has only half of the full Michelson power and thus ${\cal K}_{r,l} \to {\cal K}_{{\rm MI}}/2$. Having this in mind, one can write down the I/O-relations for the two polarization modes and for the link between them provided by the PMC unit as:

$$\begin{array}{*{20}{r}} \hfill {\left\{ {\begin{array}{*{20}{l}} {{\hat{\mathbf o}}_l} \hfill & { = {\Bbb T}_{{\rm MI}}^l{\hat{\mathbf i}}_l + {\Bbb T}_{{\rm MI}}^{b.a.}{\hat{\mathbf i}}_r + {\mathbf{t}}_l\frac{h}{{h_{{\rm SQL}}}}} \hfill \\ {{\hat{\mathbf o}}_r} \hfill & { = {\Bbb T}_{{\rm MI}}^{b.a.}{\hat{\mathbf i}}_l + {\Bbb T}_{{\rm MI}}^r{\hat{\mathbf i}}_r + {\mathbf{t}}_r\frac{h}{{h_{{\rm SQL}}}}} \hfill \\ {{\hat{\mathbf i}}_r} \hfill & { = {\Bbb P}_{\phi _0}^2{\hat{\mathbf o}}_l} \hfill \end{array}} \right.}\end{array}$$

where $${\Bbb P}_{\phi _0} = \left[ {\begin{array}{*{20}{c}} {{\mathrm{cos}}\phi _0} & { - {\mathrm{sin}}\phi _0} \\ {{\mathrm{sin}}\phi _0} & {{\mathrm{cos}}\phi _0} \end{array}} \right]$$ is the matrix for 2D rotation by angle *ϕ*_0_ that describes the phase shift the carrier light acquires as it propagates from the QWP toward the PCM, and $${\Bbb T}_{{\rm MI}}^{b.a.} = e^{2i\beta }\left[ {\begin{array}{*{20}{c}} 0 & 0 \\ { - {\cal K}/2} & 0 \end{array}} \right]$$ is the back-action-only transfer matrix of the arm that accounts for the back-action effect on the corresponding polarization mode created by the orthogonal-mode radiation pressure.

Solving these equations for ${\hat{\mathbf o}}_r$, one obtains for the new transfer matrix ${\Bbb T}[\phi _0]$ and response function ${\mathbf{t}}[\phi _0]$:

4 $$\begin{array}{*{20}{l}} {{\Bbb T}[\phi _0]} \hfill & { = {\Bbb T}_{{\rm MI}}^{b.a.} + {\Bbb T}_{{\rm MI}}^r \cdot {\Bbb P}_{\phi _0}^2 \cdot \left[ {{\Bbb I} - {\Bbb T}_{{\rm MI}}^{b.a.} \cdot {\Bbb P}_{\phi _0}^2} \right]^{ - 1} \cdot {\Bbb T}_{{\rm MI}}^l} \hfill \\ {\,{\mathbf{t}}[\phi _0]} \hfill & { = {\mathbf{t}}_r + {\Bbb T}_{{\rm MI}}^r \cdot {\Bbb P}_{\phi _0}^2 \cdot \left[ {{\Bbb I} - {\Bbb T}_{{\rm MI}}^{b.a.} \cdot {\Bbb P}_{\phi _0}^2} \right]^{ - 1} \cdot {\mathbf{t}}_l} \hfill \end{array}$$

The speed-meter regime of this interferometer is achieved when $2\phi _0 = \pi n$ for all integer *n*. In this case, one has:

5 $${\Bbb T} = - e^{4i\beta }\left[ {\begin{array}{*{20}{c}} 1 & 0 \\ { - 2{\cal K}{\mathrm{sin}}^2\beta } & 1 \end{array}} \right] = e^{2i\beta _{{\rm sag}}}\left[ {\begin{array}{*{20}{c}} 1 & 0 \\ { - {\cal K}_{{\rm sag}}/2} & 1 \end{array}} \right]$$

6 $${\mathbf{t}} = e^{2i\beta }\sqrt {\cal K} \left[ {\begin{array}{*{20}{c}} 0 \\ { - 2i{\mathrm{sin}}\beta } \end{array}} \right] = e^{i\beta _{{\rm sag}}}\sqrt {{\cal K}_{{\rm sag}}} \left[ {\begin{array}{*{20}{c}} 0 \\ 1 \end{array}} \right]$$

where ${\cal K}_{{\rm sag}} = 4{\cal K}{\mathrm{sin}}^2\beta$ is the Sagnac speed-meter OM coupling factor and $\beta _{{\rm sag}} = 2\beta + \pi /2$ is the corresponding phase shift for sidebands traveling through the Sagnac interferometer^10^. Therefore, we have shown that our scheme is equivalent to the Sagnac speed-meter interferometer with one-half the laser input power. There is no surprise in that.

And finally, substituting (5) and (6) into Eq. (3), one obtains the final expression for the PCSM quantum noise power spectral density in the form (2).

Arbitrary values of *ϕ*_0_ yield far more cumbersome formulas for ${\Bbb T}$ and **t** that one can obtain straightforwardly from Eqs. (4). However, the simple case of a small variation of *ϕ*_0_ from $\pi /2$ is of special interest for the analysis of the influence of imperfections. Let assume $\phi _0 = \pi /2 + {\it{\epsilon }}$, where ${\it{\epsilon }} = 2\pi \Delta L_{{\rm PC}}/\lambda _p \ll 1$; then one finds to first order in *ϵ*:

$${\Bbb T}_{\it{\epsilon }} = - \frac{{e^{4i\beta }}}{{1 + 2e^{2i\beta }{\it{\epsilon }}{\cal K}}}\left[ {\begin{array}{*{20}{c}} {1 + {\cal K}{\it{\epsilon }}} & { - 2{\it{\epsilon }}} \\ { - 2({\cal K}{{\sin}}^2\beta - {\it{\epsilon }})} & {1 + {\cal K}{\it{\epsilon }}} \end{array}} \right]$$

$${\mathbf{t}}_{\it{\epsilon }} = \frac{{2e^{2i\beta }\sqrt {\cal K} }}{{1 + 2e^{2i\beta }{\it{\epsilon }}{\cal K}}}\left[ {\begin{array}{*{20}{c}}{e^{i\beta }} \\ { - i{\mathrm{sin}}\beta } \end{array}} \right]$$

For phase quadrature readout, this yields the following simple expression for the QNLS PSD^18^:

7 $$S_{\it{\epsilon }}^h \simeq \frac{{h_{{\rm SQL}}^2}}{2}\left\{ {\frac{2}{{{\cal K}_{{\rm sag}}}} + \frac{{{\cal K}_{{\rm sag}}}}{2} + \frac{{2{\it{\epsilon }}\,({\cal K} - {\cal K}_{{\rm sag}})}}{{{\cal K}_{{\rm sag}}}}} \right\}$$

where the last term in the brackets dominates at low frequencies, being $\propto 1/\Omega ^6$, as we discussed above.

## Results and discussion

**Fig. 4:**
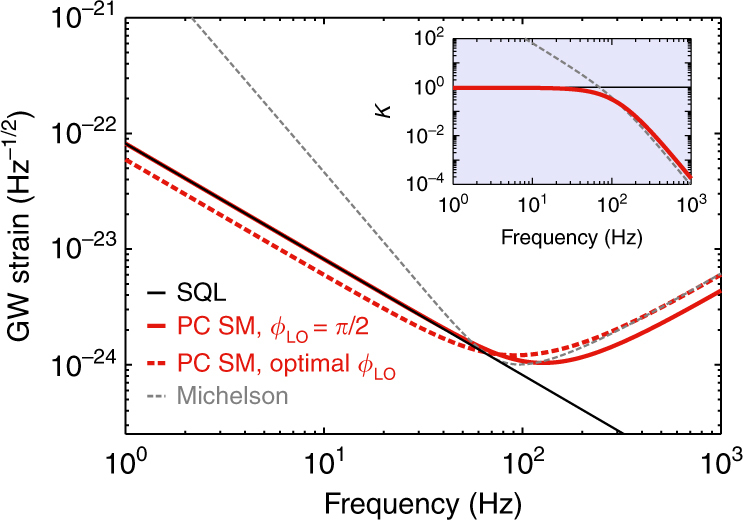
Quantum-noise-limited sensitivity (QNLS) of the polarization circulation (PC) speed meter (red traces) compared to the QNLS of the equivalent Michelson interferometer (gray dashed trace). Red dashed curve shows the potential of the speed meter to exceed the free mass SQL (black trace) in a wide band if optimal readout quadrature is measured: $\cot (\phi _{{\it{{\rm LO}}}}) = {\cal K}_{{\it{{\rm PCSM}}}}(0)$. In all other cases, we assume phase readout, i.e., $\phi _{{\it{{\rm LO}}}} = {\it{\pi }}/2$. Hereafter we assume mirrors of mass ${\it{M}} = 200$ kg, power circulating in each arm ${\it{P}}_{\it{c}} = 3$ MW, laser wavelength *λ*_0_ = 2 μm and effective interferometer bandwidth ${\it{\gamma }}/2{\it{\pi }} = 115$ Hz

The behavior of ${\cal K}$ as function of frequency reflects the strength of interaction of light with the mirrors of the interferometer at this particular sideband frequency Ω. This includes both the strength of back-action and the level of response one can expect from the particular scheme at a given signal frequency, as reflected by two terms that contain ${\cal K}$. The inset of Fig. 4 shows clearly the differences between the Michelson and the PC speed meter in this regard. The sharp rise ($\propto \Omega ^{ - 2}$) of ${\cal K}_{{\rm MI}}$ (gray trace) at low frequencies within the interferometer bandwidth, $\Omega < \gamma$, is responsible for poorer quantum noise performance of the Michelson interferometer compared to the PC speed meter, which is characterized by flat behavior of ${\cal K}_{{\rm PCSM}}$ in that frequency region. This trend is responsible for the much-improved speed-meter quantum noise at low frequencies. Moreover, as ${\cal K}_{{\rm PCSM}}(\Omega \to 0) = {\rm const}$, one can improve low-frequency sensitivity of the speed meter even more by choosing to measure the optimal quadrature by tuning the homodyne angle to $\phi _{{\rm LO}} = {\mathrm{arccot}}{\cal K}_{{\rm PCSM}}(\Omega \to 0)$, as shown by the red dashed trace in Fig. 4.

In the simple special case of $\phi _{{\rm LO}} = \pi /2$, the QNLS PSD is $S^h(\Omega ) = h_{{\rm SQL}}^2\left\{ {{\cal K} + {\cal K}^{ - 1}} \right\}/2$, and one can clearly see that ${\cal K}(\Omega _q) = 1$ is the condition of reaching the SQL. It defines the frequency *Ω*_*q*_ where the QNLS curve touches the SQL, and therefore back-action and shot noise have equal contributions to the QNLS. For the Michelson interferometer, there is always a real solution to this condition, whereas for the speed meter, there is a threshold value of the ratio $\Theta /\gamma ^3 \ge 1/4$ that sets the limit on the required circulating power for a given interferometer bandwidth and vice versa. For a given half-bandwidth *γ*, the circulating power required for the PC speed meter to reach the SQL is $P_c \ge {McL}\gamma ^3/(16\omega _p)$.

### Loss and imperfection analysis

**Fig. 5:**
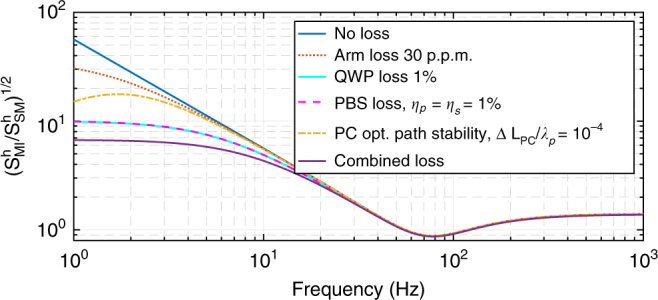
**Influences of different sources of loss and imperfection on quantum noise-limited sensitivity of the polarization circulation speed meter compared to the equivalent Michelson interferometer**

To estimate the astrophysical potential of the proposed scheme fairly, we must assess the influences of the main sources of loss and imperfections of a real interferometer. In Fig. 5, we show the relative contributions (normalized by the QNLS of the equivalent lossless Michelson interferometer) that losses and imperfections make to a realistic QNLS.

The leading source of loss for the proposed scheme is photon absorption and loss in the polarization components, i.e., absorption in the QWP (assumed single-pass photon loss of ${\it{\epsilon }}_{{\rm QWP}} = 1\%$) and loss due to imperfect polarization separation in the PBS (assumed extinction ratio for transmitted *s*-polarized and reflected *p*-polarized light of $\eta _s = \eta _p = 1\%$). One observes that both mechanisms contribute equally to the QNLS as expected because the input fields, ${\hat{\mathbf i}}_p$, pass both elements the same number of times (4) before being readout at the output as ${\hat{\mathbf o}}_p$. We also consider loss in the arm cavities, ${\it{\epsilon }}_{{\rm arm}} = 30$ ppm as a realistic projection for the next generation GW interferometers. The arm loss influence at low frequencies, as shown by Kimble et al.^17^, amounts to additional incoherent back-action noise created by loss-associated vacuum fields.

Finally, we analyze how robust the scheme is to the small deviations, $\Delta L_{{\rm PC}} \ll \lambda _p$, of the optical path length between the QWP and the PCM, defining *ϕ*_0_. Departure of *ϕ*_0_ from $\pi /2$ results in a partial leakage of the back-action term $\propto {\cal K}_{{\rm MI}}\hat i_{p,c}$ from the sine quadrature into the cosine one. This creates an additional back-action term in the quantum noise PSD $\propto 2{\cal K}_{{\rm MI}}\Delta L_{{\rm PC}}/\lambda _p \propto 1/\Omega ^4$ that leads, in conjunction with speed-meter-like response (*∝Ω*), to a steep increase of noise at low frequencies, $\sqrt {S^h} \propto 1/\Omega ^3$. This explains the downward bending of the corresponding yellow dash-dotted curve in Fig. 5 (see Eq. (7) in Materials and Methods).

To conclude this analysis, we make some remarks on balanced homodyne readout in the real scheme and the influence of laser noise and LO optical path stability on the performance of our scheme. As shown by Fritschel et al.^14^, a choice of the LO that co-propagates with the signal sidebands (e.g., pick-off at the anti-reflective coating of the main BS) solves the problems of spatial-mode mismatch and relative-phase fluctuations between the LO and the signal. Given that no modifications to the main Michelson interferometer are necessary in our scheme, the same choice of the LO is possible here with all its advantages. Steinlechner et al.^15^ showed that the effects of a DC component of the signal and path-length stability requirements for auxiliary optics that are not in the shared path can be kept at bay with the relatively modest control level of 10^−15^ m/Hz^½^ for Advanced LIGO interferometers, and therefore for our scheme as well. Finally, our scheme is not susceptible to the laser intensity noise coupling in the asymmetric Sagnac speed meters identified by Danilishin et al.^19^ for the obvious reason that a Michelson (main) interferometer such as we use here is not susceptible to BS asymmetry.

### Astrophysics results and prospects

**Fig. 6:**
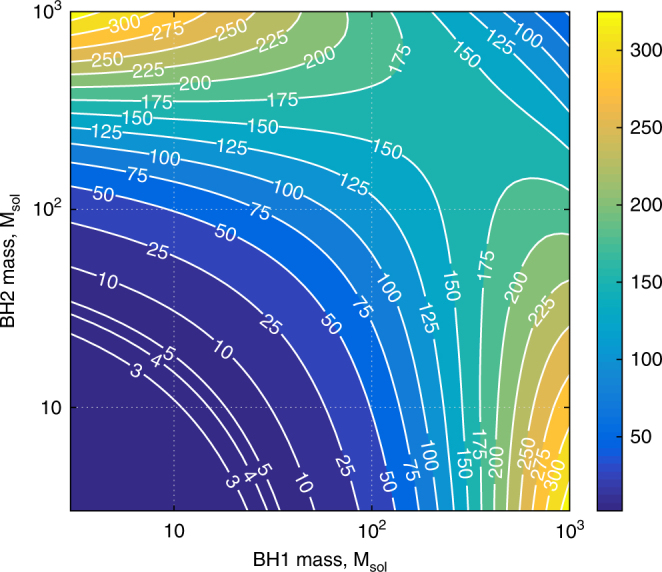
**Improvement in the anticipated rate of detection of binary black holes (BBH) coalescences (event rate) based on the amplitude spectral density of the quantum noise of the proposed scheme compared to the amplitude spectral density of the quantum noise of the equivalent Michelson interferometer**

A quantitative comparison of the QNLS of our proposed speed-meter scheme and the QNLS of an equivalent Michelson interferometer is shown in Fig. 6. (We assumed for our analysis that due to the application of enhanced techniques, all other noise sources, such as Newtonian noise^20^, seismic noise^21^, and suspension thermal noise^22^, are pushed below the level of the QNLS). For this we considered the realistic speed meter, including the optical losses shown in Fig. 5, and calculated the corresponding inspiral range (integrated for frequencies between 1 Hz and the last stable orbit), i.e., the distance up to which we can observe the BH coalescence before its signal-to-noise ratio decreases below 8. Then, we compared the speed-meter inspiral range to the inspiral range of an equivalent Michelson interferometer and derived the plotted improvement factor in terms of event rate, assuming a homogeneous source distribution throughout the Universe. We found, for example, that for initial black-hole masses similar to GW150914^1^, the speed meter would improve the event rate by a factor of ~27. The largest improvement factors, however, occur for initial black holes in the range between $100\,{\rm and}\,1000$ solar masses, achieving improvement factors larger than 100; this would allow investigating the potential existence of any intermediate-mass BH population in this, so far unobserved, mass range. Note also that although we have showcased the enabling capacity of our concept for IMBH searches, an improvement in low-frequency sensitivity inherently provides similar advantages for other GW observations, such as increasing the SNR, and for detecting BNS with better sky localization and longer warning times before their moments of merger.

## Conclusions

In this article, we suggested a new configuration for realizing a quantum speed meter in laser-interferometric GW observatories. The key advantage of our configuration compared to other speed meter implementations is that no additional optical components need to be implemented inside the main instrument. The few additional components required to convert a standard advanced GW detector into our polarization circulation speed meter can be placed in the output port of the interferometer (i.e., behind the signal-extraction cavity). Our analysis shows that compared with a standard Fabry–Perot–Michelson interferometer, our speed-meter configuration provides significantly improved sensitivity at low frequencies. Further, a detailed investigation was conducted to identify the influence of imperfections on the sensitivity. We found that the most critical factor is the optical loss of the quarter wave plate and PBS. Using realistic values for imperfections and loss, we found that the speed-meter QNLS sensitivity yielded an improvement factor of larger than 100 in the event rate for binary black-hole mergers in the range from $10^2\,{\rm to}\,10^3M_ \odot$. Future analyses will focus on further sensitivity improvements from additional complementary quantum noise reduction techniques, such as the injection of squeezed light states.
